# Identification and analysis of structurally critical fragments in HopS2

**DOI:** 10.1186/s12859-018-2551-1

**Published:** 2019-02-04

**Authors:** Sapna M. Borah, Anupam Nath Jha

**Affiliations:** Department of Molecular Biology and Biotechnology, Tezpur University, Tezpur, Assam 784028 India

**Keywords:** Modelling, Molecular dynamics, HopS2, T3SS

## Abstract

**Background:**

Among the diverse roles of the Type III secretion-system (T3SS), one of the notable functions is that it serves as unique nano machineries in gram-negative bacteria that facilitate the translocation of effector proteins from bacteria into their host. These effector proteins serve as potential targets to control the pathogenicity conferred to the bacteria. Despite being ideal choices to disrupt bacterial systems, it has been quite an ordeal in the recent times to experimentally reveal and establish a concrete sequence-structure-function relationship for these effector proteins. This work focuses on the disease-causing spectrum of an effector protein, HopS2 secreted by the phytopathogen *Pseudomonas syringae* pv. tomato DC3000*.*

**Results:**

The study addresses the structural attributes of HopS2 via a bioinformatics approach to by-pass some of the experimental shortcomings resulting in mining some critical regions in the effector protein. We have elucidated the functionally important regions of HopS2 with the assistance of sequence and structural analyses. The sequence based data supports the presence of important regions in HopS2 that are present in the other functional parts of Hop family proteins. Furthermore, these regions have been validated by an ab-initio structure prediction of the protein followed by 100 ns long molecular dynamics (MD) simulation. The assessment of these secondary structural regions has revealed the stability and importance of these regions in the protein structure.

**Conclusions:**

The analysis has provided insights on important functional regions that may be vital to the effector functioning. In dearth of ample experimental evidence, such a bioinformatics approach has helped in the revelation of a few structural regions which will aid in future experiments to attain and evaluate the structural and functional aspects of this protein family.

**Electronic supplementary material:**

The online version of this article (10.1186/s12859-018-2551-1) contains supplementary material, which is available to authorized users.

## Background

Type III secretion systems (T3SS) are specialized molecular apparatus found in gram negative bacteria which facilitate the direct injection of effector proteins into their respective animal or plant hosts [1]. These effectors display a variety of pathogenic mechanisms to take over the host machinery. The secretion systems also promote commensal or mutualistic interactions with invertebrate hosts. The molecular machinery of T3SS spans across the two layers of the bacterial membrane and helps in the extracellular adhesion of the bacteria to their respective hosts via a large extracellular appendage, known as ‘needle’ (animal pathogens) or ‘pilus’ (plant pathogens) [2–5]. The system serves as a pathway to dispense a class of proteins which are classified into two different classes. The first class known as “effectors” are released into the cytosol of the host while the other are called “translocator” or “helpers” that help in the release and function of the effectors [2, 3, 5].

*Pseudomonas syringae* pathovar tomato (Pst) strain, DC3000, is one such gram negative phyto-pathogen affecting tomato and *Arabidopsis* (in laboratory conditions). It is used as a model for exploration of the T3SS effector repository and the mechanism by which they operate in the biotrophic pathogenesis [6]. A set of 20 genes is required to encode the effectors and another set of genes for the T3SS in *P. syringae*. The avirulence (*avr*) and Hrp dependent outer protein (*hop*) genes encode the Avr and Hop proteins respectively*,* while the *Hrp* (HR and pathogenicity) and *hrc* (HR and conserved) genes encode the T3SS pathway [7, 8]. The activation of these effector proteins are regulated by the HrpL alternative sigma factor [9].

The host plant cells display a two-layered defence mechanism against these effector proteins (Hop or Avr) which also streamline the functioning of the effector proteins accordingly. The first line of defence against pathogens is the MAMP-triggered immunity (MTI) which involves the recognition of conserved Microbe-Associated Molecular Patterns (MAMPs) by pattern-recognition receptors (PRRs), which are located on the cell surface [10, 11]. The effectors’ function is mainly directed towards suppressing this MAMP-triggered immunity to favour pathogen survival and proliferation. The second line of immunity in plants includes the recognition of effectors by a special set of nucleotide-binding site leucine-rich repeat (NB-LRR) proteins which cause an Effector-Triggered Immunity (ETI) initiating a localized programmed cell death called hypersensitivity response (HR) [10, 12].

The transport mechanism of effectors through the secretion system still remains elusive. It is reported that the translocation of effector from plant pathogens through the T3SS is assisted by a signalling information that resides in the N-terminal sequence in effector proteins [7, 8]. However, there are no conserved domains reported for this N-terminal signalling region. Significantly, these regions have some salient features such as a hydrophobic amino acid at 3rd or 4th position, absence of negatively charged amino acids in the first 12 positions, and a compositional favour for serine and proline in the first 50 positions [13, 14]. A second type of signalling is assisted by type three chaperones in a manner conducive to the effective translocation of a few Hop effector proteins. These are also known as specific hop chaperones (shc). They are small (15–20 kDa), acidic and soluble [15] proteins preventing the cytoplasmic proteolysis and the premature aggregation of the effector proteins. In addition to protection, they also serve as “secretion pilots” to direct the effective translocation of the effectors through T3SS [16].

As previously mentioned, the known effector proteins do not have any conserved domain among their sequences [10] however, there are some amino acid trends which have been used in categorization as hop or avr family proteins of *P. syringae*. The reported trends include a higher serine composition, presence of an aliphatic amino acid (Ile, Leu, or Val) or Pro at the third or fourth position and absence of negatively charged amino acids in the N-terminal region [17]. However, the contribution of these features to the protein function needs to be further explored. In addition to the sequential information, some regions are identified with structural relevance. Few such evident regions are a beta motif and a probable α-helix, that assists in chaperone-effector interactions [18].

The study of effector protein family becomes a demanding task owing to the fact that the sequence similarity of these proteins is in the twilight zone. However, the availability of a few partially solved experimental structures of effectors has shed some light on the structural attributes of these proteins [19–24]. In addition to this, some structures have provided evidence on the effector-chaperone interactions. For instance, the analysis of the crystal structure of HopA1-shcA complex [21] of *P. syringae* DC3000 gives some primary features of the effector-chaperone interaction. A vital structural element namely β-motif in the effector engages with a hydrophobic patch residing in the chaperone. The β-motif is also found to be responsible for the overall stability of the complex [21]. The available effector structures provide ambiguous details about this protein family which lacks a complete overview. The structures share very less similarity and they cannot be used to predict the structure of other family members.

Experimental formulation and determination of protein structure has continued to be an indispensable field of structural biology. With the advent of state of art high performance computing these studies are now assisted by the various computational efforts to model structures for proteins. An interesting aspect is the implementation of knowledge based potentials to model proteins. These potentials are based on the amino acid interaction and its frequency of occurrence [25]. In previous studies, knowledge based potentials have been derived for amino-acids interactions in both globular and membrane proteins which can be used to model proteins [26, 27]. Homology modelling is one such knowledge-based approach in which the known structures are used as templates to obtain a model structure for sequences whose structures are not available. Whereas it is still a challenging task to predict the structure in absence of sequence similarity between the template and target sequences. It has been shown that some protein families do not possess similar structures despite having a considerable sequence similarity and performing a similar function. Such aspects are a subject of significant assessment. Molecular dynamics approach is one such technique which in blend with modelling techniques helps to  understand the time based functioning of proteins as well as protein-ligand complexes [28–31]. The technique also finds use in evaluating stability of modelled structures in a way to reveal the dynamics and functional attributes [32–34].

In this work, we have implemented an approach to recognize the structurally and functionally important regions by using different computational techniques such as ab-initio structure prediction, molecular dynamics simulation, and hydrogen bond pattern recognition. The method is applied on one such effector protein, HopS2 of *P. syringae* DC3000. This protein has a considerably strong HR suppression activity in its host [35]. A cognate chaperone shcS2 has also been reported for the efficient functioning of HopS2 [35]. The sequence based analysis has been done with an objective to highlight some salient features in terms of amino acid composition and presence of important regions in the selected protein. Subsequently, in order to furnish structural details for the protein, we have modelled HopS2 structure by two web servers and subjected each selected model for 100 ns long molecular dynamics simulations. It is observed that the regions listed from the preliminary sequence analysis are consistent during simulation and verify the importance of these regions from a structural standpoint. The elaborate understanding of these regions as functional attributes however remains a subject to experimental methods and may be used to obtain the HopS2 native structure.

## Results

### Sequence analysis of hop proteins

**Fig. 1:**
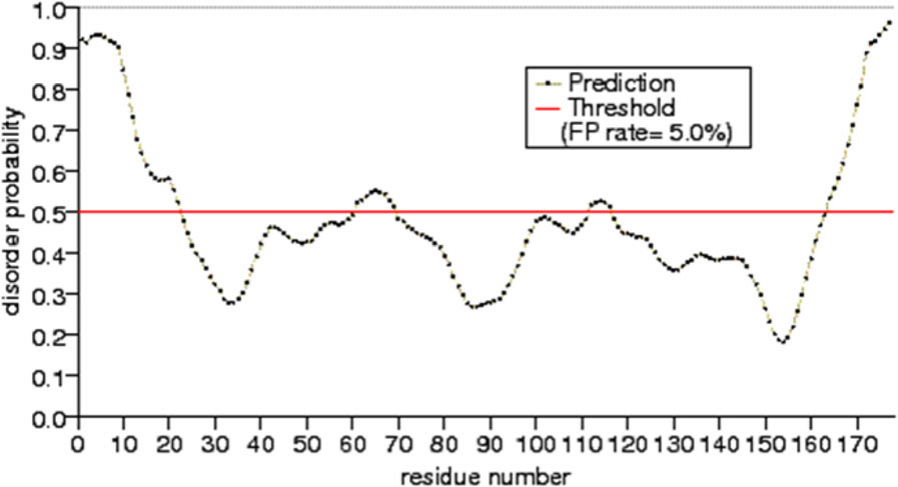
Disorder prediction by PrDOS server for HopS2

The physicochemical properties evaluated from the HopS2 protein sequence are tabulated in the Additional file 1: Table S1. A preliminary analysis of the HopS2 protein and 39 other current members of the Hop protein family do not show any conserved domain in this family, which is well reported previously. Nonetheless they are a part of the same effector repertoire and cause virulence [36]. Despite a decade of structural analysis for these effector proteins, there is trivial information that has evolved to shed significant light on these effector proteins. Most of these attempts fail at the hands of the presence of disordered regions present in these proteins [37, 38]. We have mentioned here the disordered content in HopS2 predicted from PrDOS [39] (Fig. 1). About 20% of the protein is disordered from both the N- and C- terminals, which leaves us only a probable structured region with possible chaperone interactions. Such disordered regions are also evident from previously solved X-ray structures of other effectors with most of the disorder prevailing in the N- terminal region of these proteins [15, 40, 41]. In such a scenario, any reasonable information for these proteins gathered a priori can be of considerable relevance. In light of such experimental limitations, we present here a few regions in HopS2 that can help to determine the structure of the protein. Phylogenetic analysis (data shown in Additional file 2, Figure S1) represents the relationships among the sequences which depicts that except for a few, most of the Hops are distantly related. An account of the differences in amino acids composition in HopS2 with respect to other Hop proteins is explained in supplemental file ( Additional file 3: Figure S2).

### Identification of important secondary structure regions

The experimental structure of one of the plant chaperone-effector complexes, HopA1-shcA1, reveals two key secondary structure regions that contribute to the relevant effector-chaperone interaction; the first one involves a beta motif that forms an anti-parallel beta sheet while the second one is a chaperone binding helix which inserts into the helix binding groove of the chaperone for interaction [21]. Therefore, the appearance of these vital regions in our modelled structures will help in identifying the significant structural parts contributing to the effector-chaperone interactions in HopS2. In addition to these regions, a third helical region may also be involved in chaperone interaction.

#### Evaluation of β-motif regions in HopS2

**Fig. 2:**
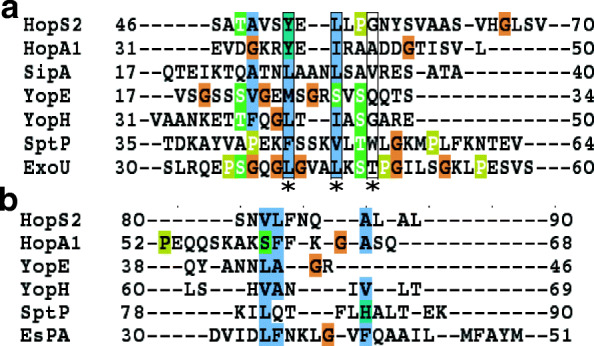
Multiple Sequence Alignment (MSA) of HopS2 **a** β-motif  region (46–70) with known structural motif in other organisms. **b** Helix 1 (80–90) with known helical regions of the PDB structures

In order to analyze the presence of these motifs in HopS2 we have initially performed a sequence based analysis. Previously, Lilic et al [15] had performed a multiple sequence alignment of a set of effector proteins and conclusively established probable β-motif regions in both plant and animal effector proteins. Some of these effectors have also shown the identified motif in their crystal structures. This directed our study towards aligning the available HopA1 and HopS2 sequence with reference to the available multiple sequence alignment data. Simultaneously, we have also retrieved six experimental structures that exhibit the corresponding chaperone-effector interaction regions; the data is enlisted in Table 1. Figure 2a represents that after alignment, the probable beta motif for HopS2 sequence spans residues 46 to 70. The beta motif residues of HopS2 that align with the rest are represented by the asterisk. All the alignments have been viewed in Jalview [42]. It is to be noted that the available PDB structures are not solved for complete sequences which limits our analysis to evidently perform full length analysis for HopS2. However, reasonable evidence from literature regarding the position of the chaperone-binding motifs has encouraged us to determine the same for HopS2.

**Table 1:** Key secondary structure regions from known effector structures

Protein name	PDB ID	β-motif	Helix 1	Helix-2
HopA1	4G6T [21]	37–41	52–67	–
SipA	2FM8 [15]	27–34	–	–
YopE	1L2W [68]	23–34	38–46	68–78
YopH	4GF3 [69]	36–46	60–69	–
SptP	1JYO [70]	45–53	78–90	130–137
EspA	1XOU [71]	–	31–59	–

#### Evaluation of chaperone-binding helical regions in HopS2

A similar alignment analysis has also been performed for the alpha helical region. The information for one of the α-helices has been retrieved from Guo et al [17], where the authors had suggested residues 82–111 of HopS2 to be α-helical and amphipathic regions that may be consistent to interact with type three secretion chaperones. The corresponding α-helical regions in other effector proteins have been retrieved from PDB structures which are consistently reported to be essentially involved in the chaperone binding interactions. The alignment has helped us to identify the region 80–90 of HopS2 having some amino acid features (Fig. 2b) similar to the functionally essential regions from the known protein structures.

### Model prediction and validation of HopS2

We initially performed modelling of the target protein by homology modelling servers like Swiss model and Phyre 2.0 which could not predict the structure for the entire sequence. The results are enclosed in the Additional file 4: Table S2. Due to the low sequence coverage in the modelled structures, we preferred *ab-inito* modelling by servers Bhageerath and Robetta that yielded 10 full length models (five from each), as shown in Additional file 5: Figure S3 (A-B). The validation of the steric quality of the models is done by Ramachandran Plot. The percentage of amino acids in the allowed region is approximately 80% in all the generated models. Additionally, a comparative study between these model structures has been done by the structural superimpositions to reveal and remove identical or very similar structures from the dataset (Additional file 6: Table S3). This will help us in streamlining our study towards structurally unique models only.

In case of Robetta, out of five predicted models, two are found to be identical (models R4 and R5). Hence, the remaining four Robetta models, R1, R2, R3 and R4 are selected. For Bhageerath, the RMSD between models B1 & B3 is 0.5 Å. Hence models B2, B3, B4 and B5 have been chosen for further evaluation. This gives altogether eight models (R1, R2, R3, R4, B2, B3, B4 and B5) with different RMSD values (> 1 Å) with approximately 90% of amino acids in favoured and allowed regions of Ramachandran plot (Additional file 7: Table S4).

The essential regions (beta motif and helices) recognised from the aforementioned sequence analysis have been checked in the modelled structures through secondary structure alignment using the STRIDE algorithm, shown in Table 2. This alignment of the modelled structures helped in identifying that majority of the models consists of the identified essential regions. This validates the occurrence of the selected secondary structural regions in HopS2 models. These local regions may be the probable binding region for its cognate chaperone shcS2 [35]. In addition to the secondary structural regions, the study has also helped in identifying a few probable beta motif residues that are prospective to interact with shcS2. Residues 52Y, 54L, 57G and others pertaining around this region of the sequence are predicted to be important for effector interactions with the cognate chaperone. The stability of these regions has been investigated by the MD simulation of the complete model structures.

**Table 2:**
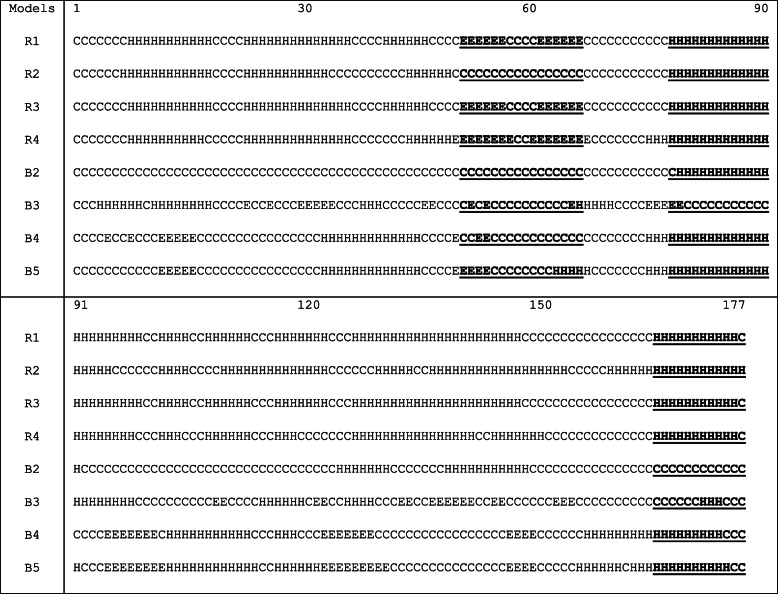
STRIDE 2D- alignment to verify the presence of the predicted secondary structure in the entire eight models. The selected amino acid regions are marked in bold and underlined

### Molecular dynamic simulation

100 ns long simulations are performed on each of the preferred eight models in order to attain a better understanding of the protein structure and function. The simulation results are discussed in the following subsections.

#### Trajectory analysis

The trajectories from the simulations are subjected to various analyses using available tools in the Gromacs package. The deviation of the models from their initial states during simulation can be evaluated using the root mean square deviation (RMSD). A higher RMSD indicates that the structure is still flexible and yet to stabilize. Comparison of the compactness of the models is done by calculating the corresponding radius of gyration (Rg) for each trajectory. A decreasing Rg generally implies that the structure is becoming more compact during the course of simulation.

**Fig. 3:**
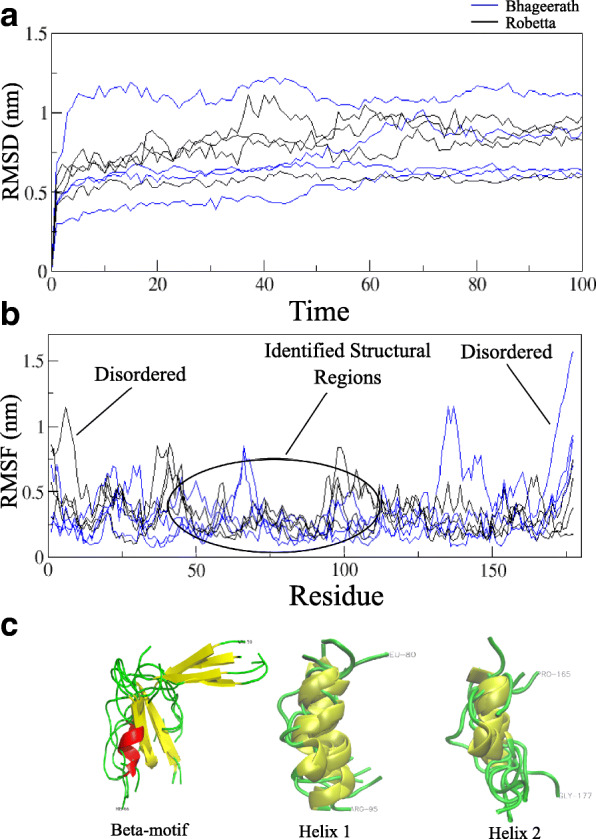
**a** RMSD as a function of time showing the structural deviations in the eight simulated models in Bhageerath (Blue) and Robetta (Black). **b** RMSF of the 8 trajectories. **c** The superimposition of the structurally critical regions in HopS2, namely the β -motif, H1 and H2

The RMSD is calculated for the modelled structures during the simulation as depicted in Fig. 3a. All the models from both the servers have attained RMSDs between a minimum and a maximum of 0.2 and 1.2 nm respectively. Details of RMSD and Rg for eight trajectories are explained in text along with the plot of Rg (Figure S4) in Additional file 8. A varied degree of RMSD may be accounted to the percentage disorder that HopS2 is predicted to hold (seen from Fig. 1). Fluctuations in the protein disordered regions, which are seen mostly as loop regions contribute to high deviations in the protein structure. A detailed analysis on the trajectory highlighting the behaviour of these residue regions is done by calculating the root mean square fluctuation (RMSF). Figure 3b depicts that the span of 50–100 residues seem to be stable with least fluctuations in the eight models altogether. This region markedly corresponds to the identified helix and beta motif region, with low probability of disorder stated by PrDOS. The N-terminal region fluctuates in most of the models owing to the greater RMSD. This is conclusive of the fact that despite the overall flexibility conferred to this protein, the local regions are stable throughout the simulation, which is also seen by the superimposition of these structural regions (Fig. 3c).

**Fig. 4:**
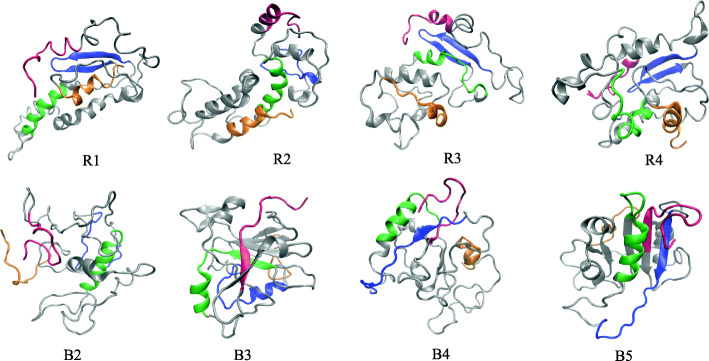
Probable β-motif and the α-helical regions have been traced in each of the simulated models. The important regions are represented in four different colours, viz., β-motif- blue; H1: green; C-terminal helix: orange; Predicted disordered regions: red

In order to present a clear picture of the spatial occurrence of the local regions identified in the previous section, we have shown the conformations of each of the eight models at the end of 100 ns simulation (Fig. 4). The relevant identified regions in Fig. 4 are coloured separately, β-motif- blue; helix-1: green; helix-2: orange. For a better view of the regions, the whole protein structures are coloured in grey. The N-terminal features a disordered signalling region for these effector proteins. We have traced the predicted N-terminal disordered regions from PrDOS in the simulated models (coloured in red) in an attempt to locate the signalling region in HopS2.

The sole intention of modelling and simulating these structures is to verify the presence of the identified regions from sequence analysis with respect to time. Figure 4 indicates that the models retain the probable beta region in five out of eight models while the helix (81–90) persists in six out of eight models, even after the long simulation time of 100 ns. It is also observed that five models represent the persistence of the previously identified regions altogether throughout the simulation period. Among the available crystal structures (Table 1), YopE and SptP represent the three secondary structural regions responsible for chaperone binding. Upon superposition of these structures with chaperone binding domains of YopE and SptP experimental structures, it is evident that two models from Robetta (R1 and R3) show closer tertiary organisation and lower pairwise RMSDs suggestive of a probable tertiary organisation in HopS2. The structural deviation observed for the models with YopE is 0.286 Å and 0.562 Å for R1 and R3 respectively. Similarly, the RMSD is 1.189 Å and 1.260 Å for R1 and R3 respectively upon superimposition with SptP.

#### Secondary structure and hydrogen bond analysis

**Fig. 5:**
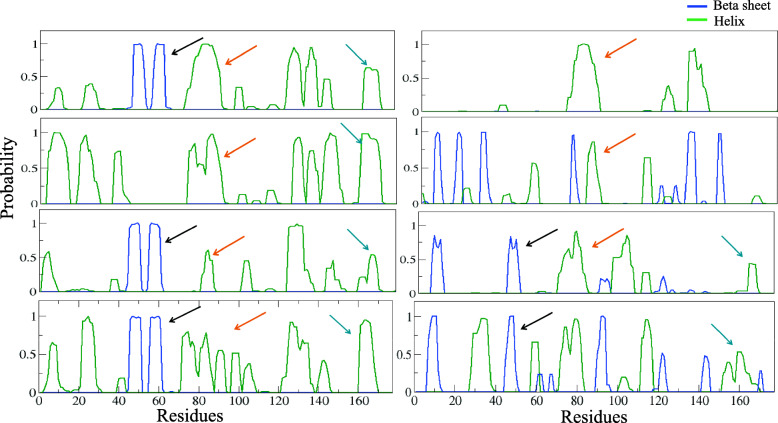
DSSP propensities in the eight simulations. One panel represent one simulation trajectory and shows the propensities of α helices and β-sheets throughout the sequence. Pointers are used to mark the probabilities for the identified regions. The pointers colours to signify: Black- β-sheets (51–66), Orange- H1 (80–90), and Blue: H2 (165–175)

DSSP has been aimed at attaining better structural insights in HopS2. Propensities for helix and beta sheet secondary structures have been calculated separately for all the eight simulations and plotted in eight different panels in Fig. 5. In each of the panels, we emphasize more on the consistency of secondary structure in the three regions possibly important for chaperone interaction. The different regions are pointed (in black, orange and blue) wherever they have occurred in the trajectory. We infer from the plot that a higher probability for the beta motif region (Fig. 5) between residues 51–66 is observed in five simulations. A similar trend is also observed for the helical (H1) region between 81 and 90 residue regions, where except for one model the rest exhibit high probabilities towards a helix formation. The probability for the C-terminal helical (H2) occurrence between residues 165–177 is seen for 6 of the models.

The secondary structure of these regions is further validated by analysing the H-bond pattern between the main chain atoms in the course of simulation. It is known that the Hydrogen bond pattern for helices is i+4/i+3 for any residue i [43]. We aim at finding the presence of such a pattern in our simulated models. The total occurrence of hydrogen bonds between the selected secondary structure regions throughout the simulation is given in Table 3. The frequency of H-bonds indicates the presence of helix in most of the simulation time in all the models. Although the length of helical region may be different in these models, the region still shows tendency to become helical along the simulation time. This not only validates the DSSP results but also suggests the occurrence of helices in 75–80% of frames. Similarly, the beta sheet occurs throughout the simulation in five of the models, converging with the DSSP results.

**Table 3:** Hydrogen Bond analysis for different selected regions

Residue pairs (Beta motif)	Occurrence (in %)	Residue pairs (H1)	Occurrence (in %)	Residue pairs (H2)	Occurrence (in %)
51–66	35	81–85	16	165–169	34
53–64	35	82–86	82	166–170	32
55–62	37	83–87	64	167–171	53
62–55	35	84–88	40	168–172	14
64–53	43	85–89	61	169–173	31
66–51	38	86–90	48	170–174	98
		87–91	37	171–175	42

### Essential dynamics

**Fig. 6:**
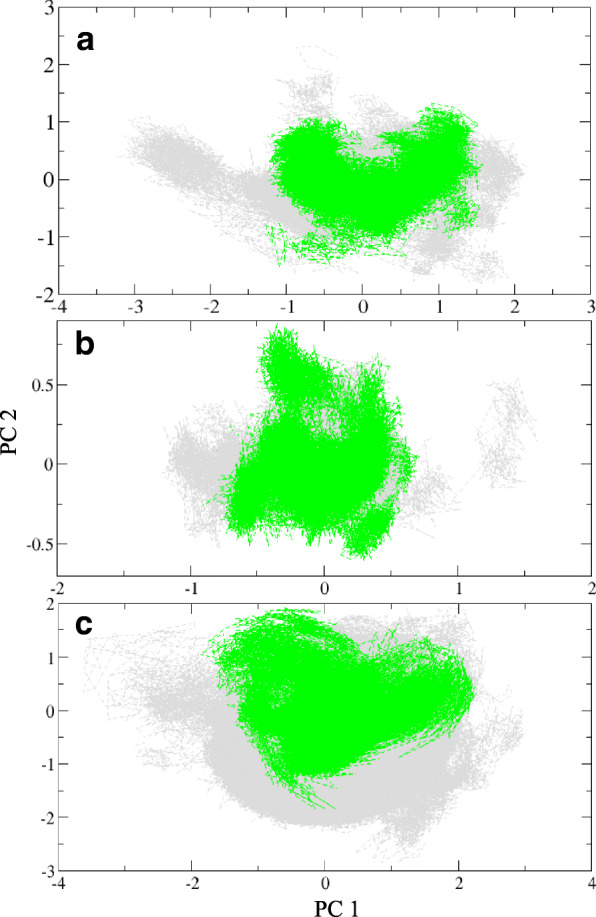
PCA for β-sheet (51–66), H1 (80–90), H2 (165–175) are shown in **a**, **b** and **c** respectively. The green and grey colours represent the density of trajectories with/without identified secondary structures

Here, we compared the stability of the identified regions in the eight individual trajectories through a principal component analysis (PCA). Only the Cα backbone atoms are included to derive the covariance and the principal components. Figure 6a-c displays the clusters for each of these regions. We performed PCA in an attempt to verify the stability of the structurally important elements; the trajectories which retain the regions are marked in green, and the contrary is painted in grey. It is seen earlier that the beta motif is retained in five among eight trajectories (from Fig. 4). The motions retrieved by the principal components in this region are recorded in Fig. 6a. Here the green subspace is quite well defined and corresponds to the models retaining the stable structural component during the course of simulation. A similar observation is accounted for the helical region which is shown in Fig. 6b. Six of the trajectories show a converged subspace as compared to the rest of the models. On the contrary, the helix 2 is not formed as clearly as the other regions. It is observed partially in three of the eight simulation trajectories, the motions of which are shown in Fig. 6c.

## Discussion

The Hop effector proteins are a family of virulent proteins with very low sequence and structural similarity among them. These proteins serve as efficient targets for the study of their pathogenicity mechanisms by which they take-over the host machinery. HopS2 is one such effector protein which stimulates a hypersensitivity response in affected plants and shares no apparent similarity with any of its family proteins. The attempts to obtain the structural information by experiments are not very successful. In this study, we have made a computational attempt to understand such effector proteins, taking HopS2 as a model. Comparison of amino acid composition reveals higher content of alanine, leucine and serine in HopS2 with respect to the average composition of other Hop family proteins. While there is no structural “fold” information in these proteins, the experimentally available effector proteins (both plant and animal pathogens) reveal secondary structure regions which are responsible for chaperone binding activity. Here, we have found that such known local structures are also reflected in the HopS2 sequence, which includes helical and a beta-motif region. Sequence based ab-initio structure prediction helped us to obtain 10 models from 2 web-servers. Eight unique models have been screened out, and further subjected to MD simulation. There is no structural convergence apparent in these models which make it difficult to comment on the global structure of HopS2. Thus, it prompted us to find the persistence of the ‘local secondary structures’ retrieved from sequence analysis. Interestingly the simulations indicate the stability of the above mentioned important secondary structural elements throughout the simulation. The validation has been done with DSSP and H-bond pattern analysis. As a result of preliminary sequence analysis and long simulations, we report about the probable stretches of residues vital to the effector protein structure and interaction. This information may be experimentally verified and further used in structural determination of these proteins. Such an analysis can be used with other members of the Hop protein family to provide introductory hints to the structure of these effector proteins which otherwise are limited by experimental techniques.

## Conclusion

MD simulation technique is a state of art computational technique that finds its way in analysis of atomistic details of biological systems. The contribution of this theoretical work is to understand relevant and possible structural regions in dearth of available information. Lack of experimental work/information on the target protein HopS2 dispenses the fact that resolving its structure has still been ineffective. In addition to this, the characterization of effector proteins and their structures needs meticulous attempts due to the presence of disordered regions that pose a great challenge to determine a protein structure. In cases as these, where the amount of information limits the knowledge pertaining to the protein, we have attempted to identify structural regions based on sequence and molecular dynamics. In an all-inclusive manner, we are reporting here a method to search and cross verify the structurally and functionally important regions for proteins that have very less sequence/structure similarity with the experimentally known protein structures. We believe that this report gives an initial idea about the structural attributes of HopS2 protein which can be applied in different experiments.

## Methods

We intend to obtain structurally and functionally important structural fragments in the HopS2 protein due to the absence of a crystallized structure. The sequence analysis followed by ab-initio structure prediction and validation has been done. Regions obtained have been studied for their stability in the model structures by long MD simulations under conditions of constant temperature and pressure. In addition to this, these regions are investigated by H-bonding pattern to identify the occurrence of important secondary structures.

### Sequence based insights of HopS2

Amino acid sequences of Hop proteins secreted by *P. syringae* DC3000 have been retrieved from Uniprot database [44] to perform a Multiple Sequence Alignment (MSA) and phylogenetic analysis using Clustal omega [45] and MEGA 5.0 [46] respectively. PrDOS has been used to predict the disorder in the protein. Different physicochemical parameters, such as molecular weight, amino acid composition, aliphatic index, etc. of HopS2 have been calculated by ProtParam server [47]. A preliminary comparative study on the complete and the N-terminal amino acid composition has been done for the selected protein with the rest of the family members.

### Identification of functional regions

In order to acquire knowledge on the functionally important regions of HopS2, we have performed a study based on the sequences and structures of the known effector proteins. A repertoire of 1215 type III secretion effectors from 221 pathogenic bacteria has been collected from the Bacterial Effector Analyzer 2.0 (BEAN 2.0) database [48]. A sequence search of these 1215 entries against the Uniprot revealed 707 active sequences while the rest being obsolete. Out of these 707, mere counts of only 56 sequences possess structural details in PDB. It is to be noted that none of these experimental structures show any significant similarity. Also due to some disordered regions that prevail in these proteins, most of the structures are not complete. The sequence length of these proteins intriguingly varies from the smallest composition of 200 amino acids to the largest being up to ~ 1900 amino acids.

To enhance our understanding about the functional attributes of HopS2, we have limited our study towards analyzing only the chaperone bound effector structures because HopS2 is also known to be functional in the presence of a cognate chaperone. The BEAN database enlists 61 such chaperone assisted hop protein sequences, amongst which 9 structures are available. It has been previously reported that certain secondary structural regions aid in chaperone-effector interactions in both animal and plant pathogens [15]. In the present study, we have tried to trace such regions in HopS2 under the consideration of the known structures.

Multiple sequence alignment is a prelude to understand local similarity in cases where lack of global identity and similarity confines our limits to retrieve information at the sequence level. In the current work, the chaperone binding domain (CBD) regions from the structures have been aligned against the entire HopS2 sequence using ClustalX [49]. Regions in HopS2 from MSA that closely facsimile CBD region from the crystal structures have been recorded for subsequent analysis.

### Model prediction of HopS2

The sequence similarity of HopS2 with other sequences in protein structure database is in the twilight zone which means that homology modelling may not be able to generate a good model structure. Swiss-model [50] and Phyre 2.0 [51] servers have been tried to generate a homology based model structure. A structure prediction method which can be applied in such a case is ab-initio structure prediction. Ab initio or de novo modelling is based on the Anfinsen’s theory of protein folding: Protein’s native structure corresponds to the state with the lowest free energy of the protein-solvent system [52]. It uses an efficient search method which can quickly identify the low-energy states through conformational search and generates a number of possible conformations. The thermodynamically stable conformations are selected as final models. Several web servers based on this theory are available which are ranked by a CASP assessment [53]. We have used two online servers, viz., Bhageerath [54] and Robetta [55] to model the 3D structures of our query protein. These two servers have been ranked amongst the top 20 from the recent CASP11 assessment in 2016 [56].

Ramachandran plot of these predicted models are obtained by using Vadar [57]. Consecutively, all the models are also superimposed with each other to procure similar sets of structures, shown in Additional file 6: Table S3. Models with a higher RMSD difference (> 1 Å) which denote unique and less similar models are selected for subsequent analysis.

### Secondary structure validation in model proteins

The sequence–structure correlation obtained from sequence alignment with PDB structures have been taken into account for validation of our modeled structures. A 2D secondary structure alignment of the HopS2 models has been done using STRIDE [58]. This aided us in analyzing the similarity of CBD secondary structures in reported proteins and in predicted models for HopS2, identified from MSA. We have tried to analyze only three major types of secondary structures which are coil (C), beta sheets (E) and helix (H).

### Molecular dynamics simulation

A set of molecular dynamics simulation is performed. Here we focus at investigating the persistence of important ‘local regions’ throughout the simulation time.

All simulations are done explicitly in Gromacs 5.0 [59], using the OPLS-AA [60] (optimized potentials for liquid simulations all atom force field). The protein models are solvated by TIP3P water model in a cubic box with periodic boundary conditions and a distance of 0.8 nm maintained between solute molecules and box edge. The system is neutralized by adding one Cl^−^ ion. All the electrostatic calculations is performed using Particle-mesh Ewald (PME) summation [61]. Energy minimization is accomplished using the steepest descent algorithm followed by equilibration of the system for 5 ns at constant pressure (1 Bar) and temperature (298 K) using Berendsen coupling [62]. The equilibrated protein models are finally subjected to a production run of 100 ns each.

The analysis of the simulation trajectories has been done using the tools available in Gromacs package. The analyses have been performed to ascertain the difference in the modeled structures along time via RMSD, RMSF and Rg. This takes into account the deviations at the secondary and the tertiary level of structures. Various snapshots are taken at different time intervals to detect these structural changes during the simulations. All the graphical visualizations are performed with VMD [63] and Pymol [64].

From each of the simulations, a total of 100 frames, at an equal interval of 1 ns, are extracted and subjected to secondary structure analysis. DSSP (Dictionary of Protein Secondary Structure) [65] method is used to study the secondary structure feature of the selected regions for the complete simulation. An average secondary structure propensity derived from 100 frames for each residue is evaluated.

The secondary structure of a protein can also be identified by observing the hydrogen bonding pattern between backbone atoms of a protein structure. Taking this into account, the selected secondary structure regions from HopS2 (from sequence analysis) is considered for H-bond pattern analysis. H-bonds has been assessed using the HBPLUS program [66]. Hydrogen bond donor and acceptor pairs are considered to identify the type of secondary structure formed in the selected regions. This is followed by analyzing the persistence of these common structural elements during the simulation of different models.

### Essential dynamics

Principal component analysis (PCA) or essential dynamics of a simulation trajectory reflects the existence of a protein conformation during a particular timeframe [67]. The dynamics of the trajectory can be initially framed in a covariance matrix from which eigen-values can be subsequently resolved. These values correspond to the principal components of a system which show the direction of motion of the trajectory. Generally, the principal components which capture approximately > 60% of the global motion in protein are considered. In this work we have extracted the essential degrees of freedom for the three identified fragments, beta motif, helix-1 and helix-2 from all the 8 trajectories for 100 separate timeframes. The global motions of these three regions are calculated by the gromacs programs, gmx covar and gmx anaeig in subsequent orders.

## Additional files

Additional file 1 **: Table S1**. Physicochemical properties of HopS2. (PDF 168 kb)

Additional file 2 **: Figure S1.** Phylogenetic analysis of Hop protein family. (PDF 115 kb)

Additional file 3 **: Figure S2:** Comparison of amino acid composition (in %) of HopS2 and Hop family sequences. The composition is represented both for the full sequence and in N-terminal (60 residues). The blue and turquoise coloured bar details the amino acid composition of full length sequence of HopS2 and 38 other Hop proteins respectively. The yellow and red bars are the N-terminal compositions of HopS2 and the averaged composition of 38 Hop proteins respectively. (PDF 126 kb)

Additional file 4 **: Table S2**. Homology prediction for HopS2. (PDF 124 kb)

Additional file 5 **: Figure S3**. Representation of the 10 predicted models from the two servers shown in (a) Robetta, (b) Bhageerath. (PDF 287 kb)

Additional file 6 **: Table S3**. Pairwise superimpositions in terms of their RMSD (Å) between all the 10 models. (PDF 118 kb)

Additional file 7 **: Table S4**. Validation of the models using Vadar. The italicized values are the validated models which are selected for subsequent simulation. (PDF 108 kb)

Additional file 8 Details of the trajectory analysis for RMSD and Rg is given in text. Figure S4. Rg for 8 simulated models. (PDF 112 kb)
