# Supplementary material for: Identification and analysis of structurally critical fragments in HopS2
Source: BMC Bioinformatics. 2019 Feb 4;19(Suppl 13):552. doi: 10.1186/s12859-018-2551-1 (PMC7394326; doi:10.1186/s12859-018-2551-1)
Supplement: Supplementary file 1 — : Table S1. Physicochemical properties of HopS2. (PDF 168 kb) [file 12859_2018_2551_MOESM1_ESM.pdf]

Table S1. Physicochemical properties of HopS2

Aliphatic index	75.71
Grand average of hydropathicity (GRAVY)	-0.201
Total number of negatively charged residues (Asp + Glu)	15
Total number of positively charged residues (Arg + Lys)	16
