# Supplementary figures and images for: Identification and analysis of structurally critical fragments in HopS2
Source: BMC Bioinformatics. 2019 Feb 4;19(Suppl 13):552. doi: 10.1186/s12859-018-2551-1 (PMC7394326; doi:10.1186/s12859-018-2551-1)

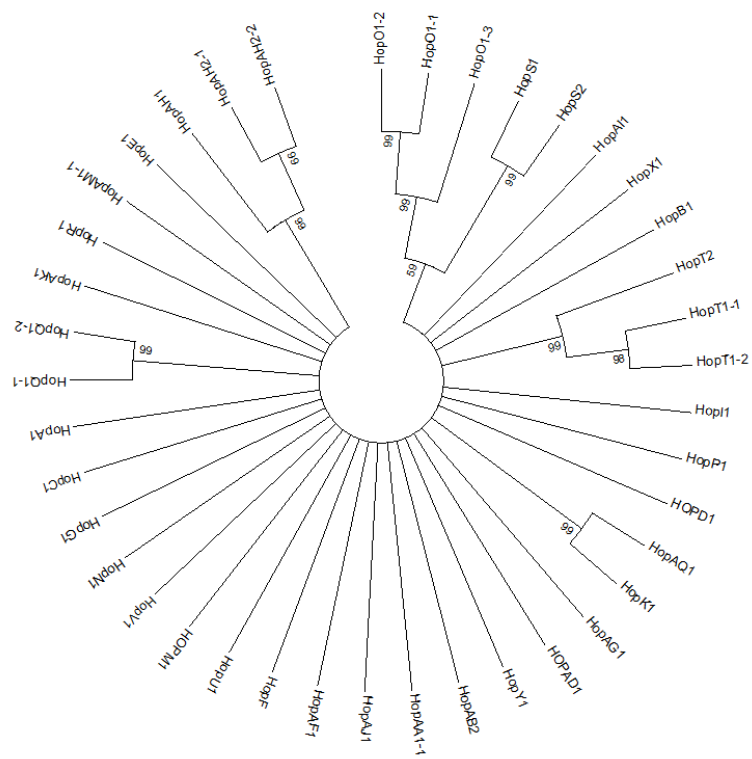

Figure S1. Phylogenetic analysis of the selected 38 Hop family sequences

Supplement: Supplementary file 2 — : Figure S1. Phylogenetic analysis of Hop protein family. (PDF 115 kb) [file 12859_2018_2551_MOESM2_ESM.pdf]
