# Supplementary material for: Identification and analysis of structurally critical fragments in HopS2
Source: BMC Bioinformatics. 2019 Feb 4;19(Suppl 13):552. doi: 10.1186/s12859-018-2551-1 (PMC7394326; doi:10.1186/s12859-018-2551-1)
Supplement: Supplementary file 3 — : Figure S2: Comparison of amino acid composition (in %) of HopS2 and Hop family sequences. The composition is represented both for the full sequence and in N-terminal (60 residues). The blue and turquoise coloured bar details the amino acid composition of full length sequence of HopS2 and 38 other Hop proteins respectively. The yellow and red bars are the N-terminal compositions of HopS2 and the averaged composition of 38 Hop proteins respectively. (PDF 126 kb) [file 12859_2018_2551_MOESM3_ESM.pdf]

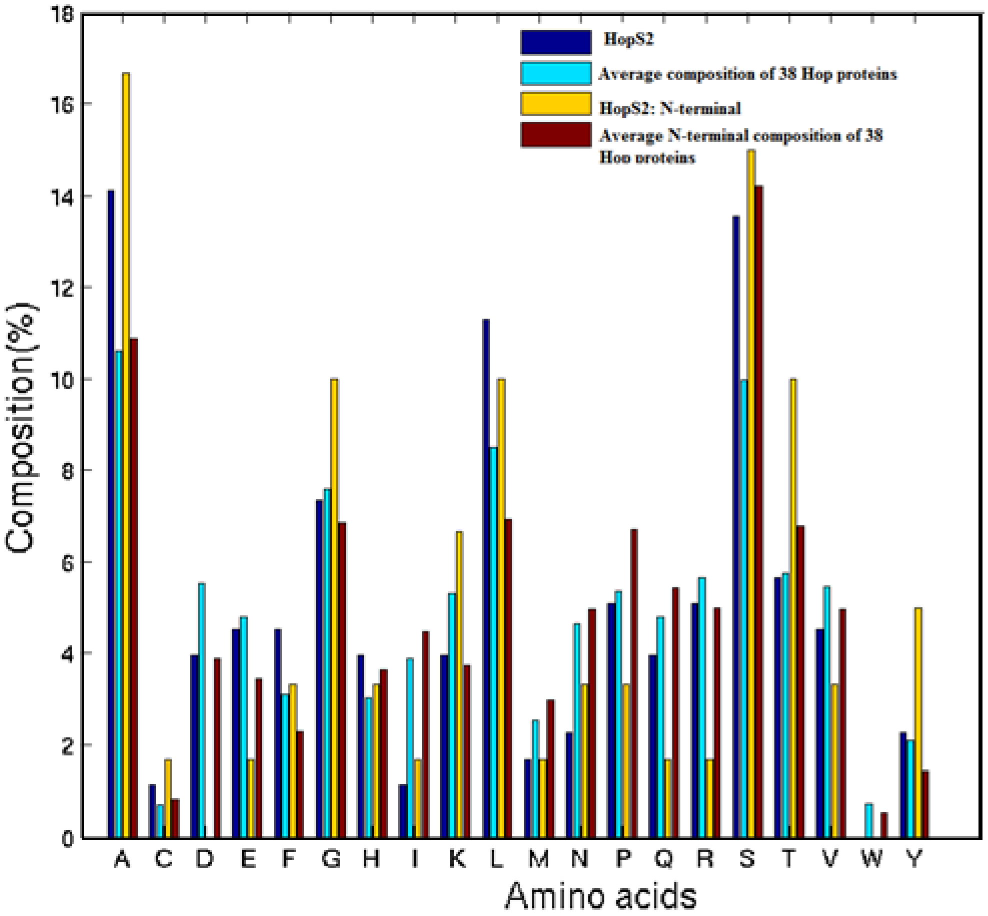

Figure S2: Comparison of amino acid composition (in %) of HopS2 and Hop family sequence. The composition is represented both for the full sequence and in N-terminal (60 residues). The blue and turquoise coloured bar details the amino acid composition of full length sequence of HopS2 and 38 other Hop proteins respectively. The yellow and red bars are the N-terminal compositions of HopS2 and the averaged composition of 38 Hop proteins respectively.
