# Supplementary material for: Identification and analysis of structurally critical fragments in HopS2
Source: BMC Bioinformatics. 2019 Feb 4;19(Suppl 13):552. doi: 10.1186/s12859-018-2551-1 (PMC7394326; doi:10.1186/s12859-018-2551-1)
Supplement: Supplementary file 4 — : Table S2. Homology prediction for HopS2. (PDF 124 kb) [file 12859_2018_2551_MOESM4_ESM.pdf]

Table S2. Homology prediction for HopS2

**Severs**	**Predicted regions**	**Predicted Structures**
Swiss-model	94-134	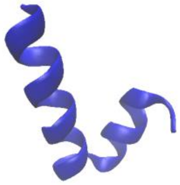
Phyre 2.0	99-126	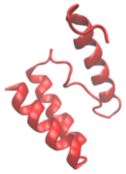
