# Supplementary material for: Identification and analysis of structurally critical fragments in HopS2
Source: BMC Bioinformatics. 2019 Feb 4;19(Suppl 13):552. doi: 10.1186/s12859-018-2551-1 (PMC7394326; doi:10.1186/s12859-018-2551-1)
Supplement: Supplementary file 5 — : Figure S3. Representation of the 10 predicted models from the two servers shown in (a) Robetta, (b) Bhageerath. (PDF 287 kb) [file 12859_2018_2551_MOESM5_ESM.pdf]

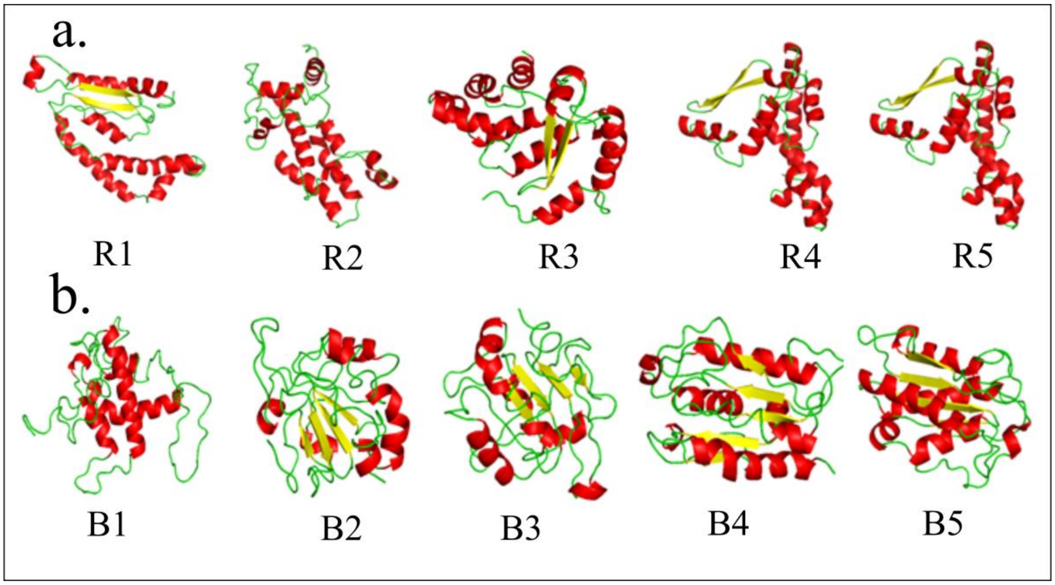

Figure S3. Representation of the 10 predicted models from the two servers shown in (a) Robetta, (b) Bhageerath.
