# Supplementary material for: Identification and analysis of structurally critical fragments in HopS2
Source: BMC Bioinformatics. 2019 Feb 4;19(Suppl 13):552. doi: 10.1186/s12859-018-2551-1 (PMC7394326; doi:10.1186/s12859-018-2551-1)
Supplement: Supplementary file 6 — : Table S3. Pairwise superimpositions in terms of their RMSD (Å) between all the 10 models. (PDF 118 kb) [file 12859_2018_2551_MOESM6_ESM.pdf]

Table S3. Pairwise superimpositions in terms of their RMSD (Å) between all the 10 models

R1	0									
R2	0.9	0								
R3	1.3	**0.9**	0							
R4	0.9	1.3	1.0	0						
R5	0.9	1.3	1.0	0	0					
B1	0.8	1.1	1.5	1.3	1.3	0				
B2	1.3	1.4	1.3	1.4	1.4	1.3	0			
B3	1.3	0.9	1.5	1.0	1.0	0.5	1.5	0		
B4	0.5	1.6	1.3	1.2	1.2	1.0	1.3	1.1	0	
B5	1.5	1.5	1.2	1.3	1.3	1.6	1.2	1.3	0.8	0
	R1	R2	R3	R4	R5	B1	B2	B3	B4	B5
