# Supplementary material for: Identification and analysis of structurally critical fragments in HopS2
Source: BMC Bioinformatics. 2019 Feb 4;19(Suppl 13):552. doi: 10.1186/s12859-018-2551-1 (PMC7394326; doi:10.1186/s12859-018-2551-1)
Supplement: Supplementary file 7 — : Table S4. Validation of the models using Vadar. The italicized values are the validated models which are selected for subsequent simulation. (PDF 108 kb) [file 12859_2018_2551_MOESM7_ESM.pdf]

Table S4: Validation of the models by Ramachandran Plot. The italicized values are the validated models which are selected for subsequent simulation analysis.

Model	Amino acids (in %)		
	Favoured	Allowed	Outliers
R1	*94.9*	*4.6*	*0.6*
R2	*92.6*	*5.7*	*1.7*
R3	93.7	*5.1*	*1.1*
R4	*95.4*	*2.9*	*1.7*
R5	95.4	2.9	1.7
B1	78.2	20.1	1.7
B2	*89.7*	*9.2*	*1.1*
B3	*82.8*	*13.8*	*3.4*
B4	*82.8*	*9.2*	*8.0*
B5	*90.9*	*4.6*	*4.6*
