# Supplementary material for: Identification and analysis of structurally critical fragments in HopS2
Source: BMC Bioinformatics. 2019 Feb 4;19(Suppl 13):552. doi: 10.1186/s12859-018-2551-1 (PMC7394326; doi:10.1186/s12859-018-2551-1)
Supplement: Supplementary file 8 — Details of the trajectory analysis for RMSD and Rg is given in text. Figure S4. Rg for 8 simulated models. (PDF 112 kb) [file 12859_2018_2551_MOESM8_ESM.pdf]

Trajectory Analysis:

1.1. Details of the trajectory analysis- RMSD and Rg:

Model R1 shows major RMSD fluctuations throughout the simulation with a maximum value of 1.2nm and attains stability after ~80ns with a value of 0.8nm. Model R2 tends to have a stable RMSD between 0.5-0.6nm and the variation is negligible after 40ns. R4 has fewer changes in the structural content after approximately 70ns with its RMSD in the order of 0.7-0.8nm. In case of the Bhageerath models, B2 is observed to deviate less after ~50ns structure with the RMSD ranging between 0.5-1.0nm. B3 shows stable RMSD after 60ns at ~0.6-0.7nm. On the other hand, B5 attains stable RMSD at ~0.5nm after ~70ns.

Rg for model R1 initially decreases up to ~10ns at around 1.8nm, and rises beyond that to about 2.1nm. However, Rg shows no major fluctuation after 60ns in the order of 2.0-2.1nm. Similarly for R2, the Rg is stable at around 1.8nm. R4, on the other hand, shows a decreasing trend in their gyration from 2.1-1.6nm till around 40ns with minor deviations after that. The models from Bhageerath have closely similar values of their radius of gyration as well. B2 acquires a stable Rg decreasing from 2 to ~1.6nm. Rg for B3 increases minutely from ~1.5nm in the initiation of the simulation, and remains in the order of 1.5-1.6nm for the entire simulation time. Similarly, B5 also has stable Rg values at approximately 1.5nm throughout the simulations.

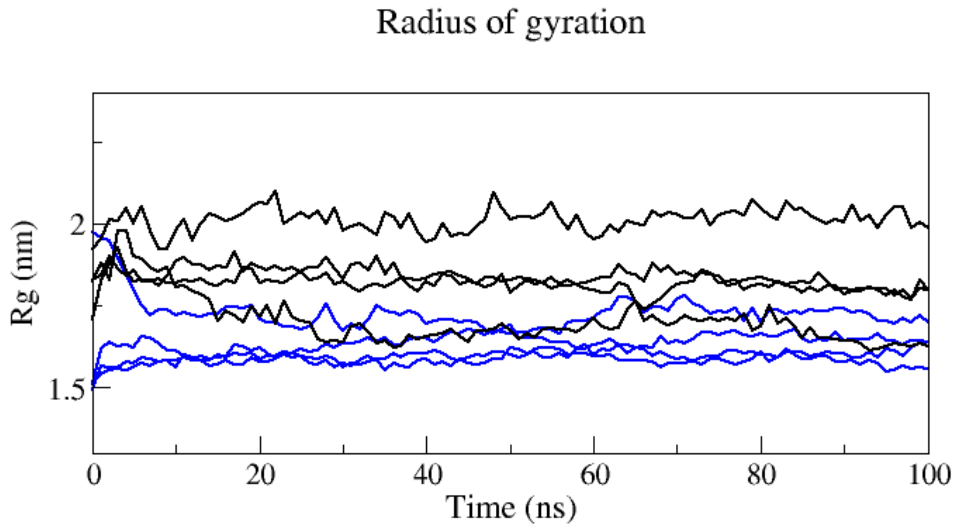

Figure S4. Radius of gyration for eight simulations
